# Supplementary material for: The Evolution and Characterization of the RNA Interference Pathways in Lophotrochozoa
Source: Genome Biol Evol. 2024 May 7;16(5):evae098. doi: 10.1093/gbe/evae098 (PMC11114477; doi:10.1093/gbe/evae098)
Supplement: evae098_Supplementary_Data [file evae098_supplementary_data.zip › Supplementary_materials.zip!/selected_miRNAs/NoGe_selected_miRNAs/mirdeep_pdf_files/NMRB01000688.1_Notospermus_geniculatus_strain_Ushimado_scaffold688,_whole_genome_shotgun_sequence_18801.pdf]

Provisional ID	:	NMRB01000688.1_Notospermus_geniculatus_strain_Ushimado_scaffold688,_whole_genome_shotgun_sequence_18801
Score total	:	2443.3
Score for star read(s)	:	3.9
Score for read counts	:	2438.1
Score for mfe	:	1.9
Score for randfold	:	0
Score for cons. seed	:	-0.6
Total read count	:	4794
Mature read count	:	4442
Loop read count	:	0
Star read count	:	352

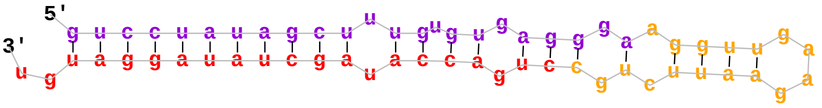

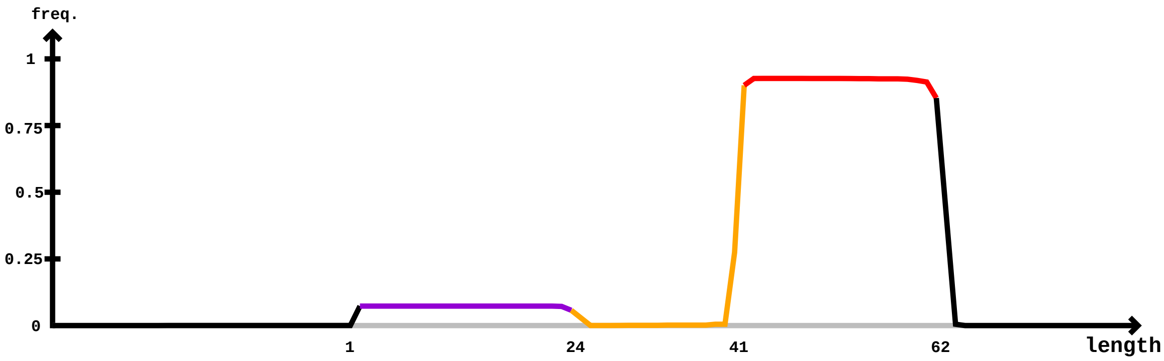

Star   Mature

```
5'- cugagucugcaagagaggcaagucuugggguguccuauagcuuugugugagggaagguugaagaaauucugccugaccauagcuauaggauguccaucucuuguucacacauc  -3'  obs
    cugagucugcaagagaggcaagucuugggguguccuauagcuuugugugagggaagguugaagaaauucugccugaccauagcuauaggauguccaucucuuguucacacauc       exp
    ....((..((((((((.........((((((((((((((((.((.((.(((.(.((((....)))).).))).)))).))))))))))))))))))))))))..))..... reads mm sample
    ..............................guccuauagcuuugugAgagg.............................................................   1     1   Fm1
    ..............................guccuauagcuuugugugagg.............................................................   4     0   Fm1
    ..............................guccuauagcuuuguguUaggg............................................................   1     1   Fm1
    ..............................guccuauagcuuugugugaggg............................................................   6     0   Fm1
    ..............................guccuauagcuuugugugagggG...........................................................   2     1   Fm1
    ..............................guccuauagcuuugugugagggC...........................................................   9     1   Fm1
    ..............................guccuauagcuuugugugaggCa...........................................................   1     1   Fm1
    ..............................guccuauagcuuugugGgaggga...........................................................   2     1   Fm1
    ..............................guccuauagcuuugugugaggga...........................................................   68    0   Fm1
    ..............................guccuauagcuuuguguAaggga...........................................................   1     1   Fm1
    ..............................guccuauagcGuugugugaggga...........................................................   1     1   Fm1
    ...............................................................aauucugccugaccauagcuaA...........................   4     1   Fm1
    ....................................................................ugccugaccauagcuauaggaug.....................   3     0   Fm1
    ....................................................................ugccugaccauagcuauaggauU.....................   2     1   Fm1
    ......................................................................ccugaccauagcuauagg........................   4     0   Fm1
    ......................................................................ccugaccauagcuauaUgaug.....................   1     1   Fm1
    ......................................................................ccugaccauagcuauaCgaug.....................   1     1   Fm1
    ......................................................................ccugaccauagcuauagCaug.....................   1     1   Fm1
    ......................................................................ccugaccauagcuauaggaug.....................   9     0   Fm1
    ......................................................................ccugaccauaUcuauaggaug.....................   1     1   Fm1
    ......................................................................ccugaccauagcuauagUaugu....................   1     1   Fm1
    ......................................................................ccugaccauagcuGuaggaugu....................   1     1   Fm1
    ......................................................................ccugaccauagcuauaggaugu....................   239   0   Fm1
    ......................................................................Ncugaccauagcuauaggaugu....................   1     1   Fm1
    ......................................................................ccugaccauagcuauaggUugu....................   1     1   Fm1
    ......................................................................cAugaccauagcuauaggaugu....................   1     1   Fm1
    ......................................................................ccugaccauagcuauagAaugu....................   3     1   Fm1
    ......................................................................ccugaccauaAcuauaggaugu....................   2     1   Fm1
    ......................................................................ccugaccauagcuauagCaugu....................   2     1   Fm1
    ......................................................................ccugaccauagcuauaggaugC....................   2     1   Fm1
    ......................................................................ccugaccauagcuauaggauguU...................   97    1   Fm1
    ......................................................................ccugaccauagcuauaggauguA...................   7     1   Fm1
    .......................................................................cugaccauagcuauagga.......................   17    0   Fm1
```

Star **Mature**

cugagucugcaagagaggcaagucuuggguguccuauagcuuugugugagggaagguugaagaauucugccugaccauagcuauaggauguccaucucuuguucacacauc			
....cugaccauagcuauaggaA......................	3	1	Fm1
....cugaccauagcuauaggau......................	7	0	Fm1
....cugaccauagcuauaggCug.....................	1	1	Fm1
....cugaccauagcuauagCaug.....................	1	1	Fm1
....cugaccauagcuauaggaug.....................	57	0	Fm1
....cugaccauCgcuauaggaugu....................	1	1	Fm1
....cugaccauagcuauagAaugu....................	2	1	Fm1
....cugaccauUgcuauaggaugu....................	2	1	Fm1
....cugaccauagcuauaggCugu....................	2	1	Fm1
....cugaccauagcuauaggUugu....................	5	1	Fm1
....cugaccauagcuauaggaugG....................	7	1	Fm1
....cugaccauagcuauaggGugu....................	2	1	Fm1
....cugaccauGgcuauaggaugu....................	1	1	Fm1
....cAgaccauagcuauaggaugu....................	1	1	Fm1
....cugaccauagAuauaggaugu....................	3	1	Fm1
....cugaccauagcuGuaggaugu....................	1	1	Fm1
....cugaccauagcuauagCaugu....................	1	1	Fm1
....cugaccauagcuauaggaugu....................	741	0	Fm1
....cCgaccauagcuauaggaugu....................	2	1	Fm1
....cugaccauagcuauaggauguU...................	188	1	Fm1
....cugaccauagcuauaggauguc...................	4	0	Fm1
....cugaccauagcuauaggauguA...................	30	1	Fm1
....cugaccauagcuauaggauguAc..................	2	1	Fm1
....ugaccauagcuauaggaug.....................	4	0	Fm1
....ugaccauagcuauaggaugu....................	4	0	Fm1
....ugaccauagcuauaggauguA...................	2	1	Fm1
....ugaccauagcuauaggauguU...................	12	1	Fm1
....ugaccauagcuauaggauguUc..................	1	1	Fm1
....ugaccauagcuauaggaugucU..................	3	1	Fm1
..............................guccuauagcuuugugGgaggga..	1	1	Fm2
..............................guccuauagcuuugugugaggga..	42	0	Fm2
..............................guccuauagcuuuguguAaggga..	2	1	Fm2
....ccugaccauagcuauaggaug.....................	1	0	Fm2
....ccugaccauagAuauaggaugu....................	1	1	Fm2
....ccugaccauagcuauaggaugu....................	61	0	Fm2
....cAugaccauagcuauaggaugu....................	1	1	Fm2
....ccugaccauagcuauagAaugu....................	1	1	Fm2
....Ncugaccauagcuauaggaugu....................	1	1	Fm2
....ccugaccauagcuauaggauguU...................	21	1	Fm2
....cugaccauagcuauaggau......................	2	0	Fm2
....cugaccauagcuauaggaug.....................	30	0	Fm2
....cugaccauagcuauaggaugG....................	2	1	Fm2
....Gugaccauagcuauaggaugu....................	1	1	Fm2
....cugaccauagcuauaggaugu....................	255	0	Fm2
....cugaccauagcuauagCaugu....................	1	1	Fm2
....cugaccauagcuauaggauguA...................	5	1	Fm2
....cugaccauagcuauaggauguU...................	48	1	Fm2
....cugaccauagcuauaggauguUc..................	14	1	Fm2
....ugaccauagcuauaggaCgu....................	1	1	Fm2
....ugaccauagcuauaggaugu....................	3	0	Fm2
..............................guccuauagcuuugugugagg..	1	0	Mm2
..............................guccuauagcuuugugugaggg...	3	0	Mm2
..............................guccuauagcuuuAugugaggg...	1	1	Mm2
..............................guccuauagcuuuguguAaggga..	1	1	Mm2
..............................guccuauagcuuugugCgaggga..	1	1	Mm2
..............................guccuauagcuuugugAgaggga..	1	1	Mm2
..............................guccuauagcuuugugugaggga..	51	0	Mm2
....aagguugaagaauucugccugaccU..................................	1	1	Mm2
....gaagaauucugccugaccauagc..............................	1	0	Mm2
....gaagaauucugccugaccauagcu.............................	1	0	Mm2
....ccugaccauagcuauaggau......................	1	0	Mm2
....ccugaccauagcuauaggaug.....................	4	0	Mm2
....ccugaccauagcuauagUaugu....................	1	1	Mm2
....ccugaccauagcuauaggaugu....................	108	0	Mm2
....ccugaccauagcuauaggauguU...................	25	1	Mm2
....ccugaccauagcuauaggauguA...................	1	1	Mm2
....cugaccauagcuauaggau......................	1	0	Mm2
....cugaccauagcuauaggaug.....................	2	0	Mm2

Star Mature

cugagucugcaagagaggcaagucuugggu**guccuauagcuuugugugaggga**agguugaagaauucugc**cugaccauagcuauaggaugu**ccaucucuuguucacacauc			
....cugaccauagcuauaggaugC....................	1	1	Mm2
....cugaccauagcuauaggaugG....................	2	1	Mm2
....cugaccauagcuauaggaugu....................	97	0	Mm2
....cugaccauagcuauaggauguc...................	1	0	Mm2
....cugaccauagcuauaggauguU...................	39	1	Mm2
....cugaccauagcuauaggauguA...................	5	1	Mm2
....ugaccauagcuauaggaugu....................	1	0	Mm2
....ugaccauagcuauaggauguU...................	3	1	Mm2
....ugaccauagcuauaggauguA...................	4	1	Mm2
....gaccauagcuauaggauguU...................	1	1	Mm2
..............................guccuauagcuuugugAgaggg...	1	1	Mm1
..............................guccuauagcuuugugugaggg...	21	0	Mm1
..............................guccuauagcuuugCgugaggg...	1	1	Mm1
....ccugaccauagcuauaggaugu....................	89	0	Mm1
....ccugaccauagcuauagCaugu....................	1	1	Mm1
....ccugaccauagcuauaCgaugu....................	2	1	Mm1
....cugaccauagcuauagUaugu....................	1	1	Mm1
....cCgaccauagcuauaggaugu....................	9	1	Mm1
....cugaccauagcuauaggUugu....................	1	1	Mm1
....cugaccauagcuauagCaugu....................	1	1	Mm1
....cugaccauagcuauaggaugG....................	1	1	Mm1
....cugaccauagcuauaggaugu....................	334	0	Mm1
....cugaccauagcuauaggauguU...................	14	1	Mm1
....cugaccauagcuauaggauguA...................	8	1	Mm1
..........aagagaggcaagucuugggu...	1	0	Fm3
..............................gucUuauagcuuugugugaggg...	1	1	Fm3
..............................guccuauagcuuuguguAaggg...	1	1	Fm3
..............................guccuauagcuuugugugaggg...	28	0	Fm3
..............................guccuauagcuuugugugaggU...	2	1	Fm3
..............................guccuauagcuuugugugaggga..	69	0	Fm3
..............................guccuauagcuuugugGgaggga..	1	1	Fm3
..............................guccuauagcuuugugugagggaa...	2	0	Fm3
....ugaagaauucugccugaccauagcu.............................	1	0	Fm3
....ugccugaccauagcuauaggaug.....................	10	0	Fm3
....ccugaccauagcuauagg........................	2	0	Fm3
....ccugaccauagcuauagga.......................	1	0	Fm3
....ccugaccauagcuauaggau......................	6	0	Fm3
....ccugaccauUgcuauaggaug.....................	1	1	Fm3
....ccugaccauagcuauaggaug.....................	63	0	Fm3
....ccugaccauagcuauaAgaug.....................	1	1	Fm3
....ccugaccauaCcuauaggaug.....................	1	1	Fm3
....ccuAaccauagcuauaggaugu....................	1	1	Fm3
....ccugaccauaUcuauaggaugu....................	1	1	Fm3
....ccugaccauagcuGuaggaugu....................	1	1	Fm3
....ccGgaccauagcuauaggaugu....................	1	1	Fm3
....ccugaccauagcuauaAgaugu....................	1	1	Fm3
....ccCgaccauagcuauaggaugu....................	1	1	Fm3
....ccugacUauagcuauaggaugu....................	1	1	Fm3
....ccugaccauagcuauagAaugu....................	2	1	Fm3
....ccugaccauagcuauaUgaugu....................	2	1	Fm3
....ccugaccauagcuauagCaugu....................	2	1	Fm3
....ccugaccauagcuauaggaugu....................	357	0	Fm3
....ccugaccauagcuauaggauAu....................	1	1	Fm3
....ccugaccauagcuauaggauguA...................	5	1	Fm3
....ccugaccauagcuauaggauguU...................	63	1	Fm3
....cugaccauagcuauagga.......................	4	0	Fm3
....cugaccauagcuauaggau......................	6	0	Fm3
....cugaccauagcuauaggaug.....................	70	0	Fm3
....cuAaccauagcuauaggaug.....................	1	1	Fm3
....cCgaccauagcuauaggaugu....................	1	1	Fm3
....Augaccauagcuauaggaugu....................	1	1	Fm3
....cugaccauagcuauaggGugu....................	5	1	Fm3
....cugaccauagcuauaggUugu....................	2	1	Fm3
....cAgaccauagcuauaggaugu....................	1	1	Fm3
....cugaccauagcuauaggCugu....................	1	1	Fm3
....Uugaccauagcuauaggaugu....................	1	1	Fm3
....cugaccauagAuauaggaugu....................	2	1	Fm3
....cugaccaCagcuauaggaugu....................	1	1	Fm3

Star **Mature**

```
cugagucugcaagagaggcaagucuuggguguccuauagcuuugugugagggaagguugaagaauucugccugaccauagcuauaggauguccaucucuuguucacacauc
......................................................................cugaccauagcuauaggaugA....................    3    1   Fm3
......................................................................cugaccauagcuauaggaugu....................  591    0   Fm3
......................................................................cugaccauUgcuauaggaugu....................    1    1   Fm3
......................................................................cugacUauagcuauaggaugu....................    2    1   Fm3
......................................................................cugaccauagcuauagCaugu....................    3    1   Fm3
......................................................................cugaAcauagcuauaggaugu....................    1    1   Fm3
......................................................................cugaccauagcuauagUaugu....................    2    1   Fm3
......................................................................cugaccauCgcuauaggaugu....................    1    1   Fm3
......................................................................cGgaccauagcuauaggaugu....................    1    1   Fm3
......................................................................cugaccauagcuaCaggaugu....................    1    1   Fm3
......................................................................cugaccauagcuauGggaugu....................    1    1   Fm3
......................................................................cugaccauagcuauaggauguc...................    4    0   Fm3
......................................................................cugaccauagcuauaggauguA...................   35    1   Fm3
......................................................................cugaccauagcuauaggauguU...................  185    1   Fm3
......................................................................cugaccauagcuauaggaugucU..................    3    1   Fm3
.......................................................................ugaccauagcuauaggaCg.....................    1    1   Fm3
.......................................................................ugaccauagcuauaggaug.....................   16    0   Fm3
.......................................................................ugaccauagcuauaggaGgu....................    1    1   Fm3
.......................................................................ugaccauagcuauaggaugu....................   19    0   Fm3
.......................................................................ugaccauagcuauaggauguA...................   19    1   Fm3
.......................................................................ugaccauagcuauaggauguU...................   27    1   Fm3
.......................................................................ugaccauagcuauaggauguAc..................    1    1   Fm3

..............................guccuauagcuuugugugaggU...........................................................    2    1   Mm3
..............................guccuauagcuuugugugaggg...........................................................    3    0   Mm3
..............................guccuauagcuuugugugaggga..........................................................   18    0   Mm3
..............................guccuauagcuuugugCgaggga..........................................................    1    1   Mm3
..............................guccuauagcuuuguguAaggga..........................................................    1    1   Mm3
...................................................................ugccugaccauagcuauaggaug.....................    1    0   Mm3
.....................................................................ccugaccauagcuauaggau......................    1    0   Mm3
.....................................................................ccugaccauagcuauaggaug.....................    4    0   Mm3
.....................................................................ccugaccauagcuauaggaugu....................   72    0   Mm3
.....................................................................ccugaccauaCcuauaggaugu....................    1    1   Mm3
.....................................................................ccugaccauagcuauagAaugu....................    2    1   Mm3
.....................................................................ccugaccauagcuauagCaugu....................    1    1   Mm3
.....................................................................ccugaccauagcuauaggauguU...................   10    1   Mm3
.....................................................................ccugaccauagcuauaggauguA...................    1    1   Mm3
......................................................................cugaccauagcuauaggau......................    2    0   Mm3
......................................................................cugaccauagcuauaggaug.....................    3    0   Mm3
......................................................................cugaccauagcuauaggCugu....................    1    1   Mm3
......................................................................cugaccauagcuauaggGugu....................    1    1   Mm3
......................................................................cugaccauagcuauagCaugu....................    1    1   Mm3
......................................................................cugaccauagcuauaggaugu....................  105    0   Mm3
......................................................................cugaccauagcuauaggauguU...................   17    1   Mm3
......................................................................cugaccauagcuauaggauguA...................    2    1   Mm3
.......................................................................ugaccauagcuauaggaugu....................    1    0   Mm3
.......................................................................ugaccauagcuauaggauguA...................    1    1   Mm3
```
